# LncRNA NEAT1 Targets miR-342-3p/CUL4B to Inhibit the Proliferation of Cutaneous Squamous Cell Carcinoma Cells

**DOI:** 10.1155/2022/8145129

**Published:** 2022-07-22

**Authors:** Zhenhua Gong, Yi Zhang, Yasu Jiang, Peng Chen, Jianfeng Ji

**Affiliations:** ^1^Department of Burn and Plastic Surgery, Affiliated Hospital 2 of Nantong University, The First People's Hospital of Nantong, Nantong, China; ^2^Nantong Clinical Medical College, Kangda College of Nanjing Medical University, Nantong, China; ^3^Department of Neurosurgery, Affiliated Hospital 2 of Nantong University, The First People's Hospital of Nantong, Nantong, China

## Abstract

**Objective:**

This study investigated whether lncRNA NEAT1 could inhibit the proliferation of cutaneous squamous cell carcinoma (CSCC) cells by targeting miR-342-3p/CUL4B, thereby affecting the occurrence and development of CSCC.

**Methods:**

Fluorescence quantitative PCR was used to detect the expression of lncRNA NEAT1 and miR-42-3p in skin squamous cell carcinoma and adjacent tissues. Bioinformatics software and luciferase reporter gene assay were used to analyze the association of lncRNA NEAT1 and miR-342-3p. The effect of overexpression or knockdown of miR-342-3p on the proliferation of CSCC cells was examined by MTT and colony formation assays. Western blotting was used to detect the proteins of the miR-342-3p/CUL4B signaling axis.

**Results:**

The lncRNA NEAT1 is abnormally overexpressed in CSCC tissues and cell lines. The expression of lncRNA NEAT1 and miR-342-3p in CSCC was negatively correlated. Bioinformatics prediction analysis revealed that lncRNA NEAT1 regulates the expression of miR-342-3p. The results of MTT and plate colony formation experiments showed that the transfection of miR-342-3p mimics significantly inhibited the proliferation and plate colony formation of CSCC cells, while the transfection of miR-342-3p inhibitor significantly promoted the proliferation and plate colony-forming ability of CSCC cells. Western blot results showed that lncRNA NEAT1 affected CSCC cell proliferation through miR-342-3p/CUL4B/PI3K-Akt signaling pathway.

**Conclusion:**

The expression of lncRNA NEAT1 and miR-342-3p in CSCC tissues was negatively correlated. This study is the first to demonstrate that the lncRNA NEAT1, as a ceRNA, affects the proliferation of skin squamous cell carcinoma cells through the miR-342-3p/CUL4B/PI3K-Akt signaling pathway. Therefore, lncRNA NEAT1 could be a biological marker or target for CSCC diagnosis or treatment.

## 1. Introduction

In recent years, the incidence of skin cancer is increasing worldwide [1]. Nonmelanoma skin cancer accounts for ∼95% of the total number of skin cancers including malignant melanoma skin cancer, basal cell carcinoma (BCC), and cutaneous squamous cell carcinoma (CSCC) [2, 3]. Keratinocytes are the primary cause of CSCC, which is the second most frequent nonmelanoma skin cancer. The development of CSCC occurs rapidly which is the fundamental reason for death from nonmelanoma cancers [4]. Since the early manifestations of CSCC are not typical and difficult to diagnose, most patients often formed invasive CSCC when diagnosed clinically [5]. Lymph node metastasis is reported in 5% of cases with CSCC with tumors that may have spread to the viscera. Patients with lymph node or distant metastases have a 10-year survival rate of fewer than 20%, showing that the treatment of advanced and metastatic CSCC is a major challenge [4]. Therefore, the molecular mechanism of CSCC occurrence and development should be further studied to find new diagnostic and therapeutic targets to inhibit tumor invasion and metastasis and improve the therapeutic effect.

One of the main classes of noncoding RNAs is lncRNA, which has over 200 bases in length. Similar to coding genes, lncRNAs can be extensively involved in biological, developmental, and pathological processes utilizing chromosome recombination, enhancer cis-element, and posttranscriptional regulation [6, 7]. LncRNA NEAT1 is a recently identified unspliced polyadenylate noncoding transcript from a human 11q13 locus known as multiple endocrine tumor type I by RNA polymerase II [8]. Recent investigations have proved that lncRNA NEAT1 is tightly linked to the growth, metastasis, and proliferation of various tumors. For example, lncRNA NEAT1 promotes breast cancer growth by controlling miR-410-3p/CCND1 axis, demonstrating the therapeutic potential of lncRNA NEAT1 [9]. This finding indicates that lncRNA NEAT1 is capable of inducing the progression of breast cancer. As colorectal cancer development and metastasis are facilitated by the stimulation of the Wnt signaling pathway activated through lncRNA NEAT1, the lncRNA NEAT1/DDX5/Wnt/-catenin axis might be an effective therapeutic target [10]. Knockdown of lncRNA NEAT1 was demonstrated to significantly decrease the proliferation and invasion of CSCC [11]. However, the mechanism by which lncRNA NEAT1 controls cell proliferation and invasion is still unknown. According to the preliminary findings of bioinformatics study, the lncRNA NEAT1 is found in the cytoplasm. This lncRNA has the potential to act as a competitive endogenous RNA (ceRNA) and regulate the expression of target genes. Furthermore, by directly controlling the miRNAs located within the cell, the lncRNA plays an important role in regulating the genesis for the progression of malignancies.

The aim of this study is to investigate the role of lncRNA NEAT1 in CSCC proliferation and its relation to the miR-342-3p/CUL4B axis.

## 2. Materials and Methods

### 2.1. Clinical Sample Collection

In total, 20 cases of cancer tissues and paired para-cancerous normal tissues of patients who underwent surgical treatment for skin cancer from January 2020 to December 2020 were selected. The Ethics Committee of the affiliated hospital 2 of Nantong university approved the study. All participants signed the inform letters.

### 2.2. Cell Culture

Shanghai Institute of Biological Sciences provided the human normal skin HaCaT cell and the CSCC cell lines including A431, SCL-1, SCC13, and HSC-5. The cells were grown in an RPMI-1640 medium that included 100 *μ*g/ml streptomycin (Shanghai Guchen Biological Co., LTD, China), 100 U/mL penicillin (Shanghai Guchen Biological Co., LTD, China), and 10% fetal bovine serum (Sigma-Aldrich, St Louis, MO). The cells were incubated at 37°C with 5% CO_2_.

### 2.3. Real-Time Fluorescence Quantitative PCR (qRT-PCR)

Trizol (Invitrogen, USA) was used to isolate total RNA from various cell types and tissue types. Following the RNA extraction process, the total RNA concentration was determined by the use of Nanodrop2000 (Thermo Scientific, USA), and the level of expression for lncRNA NEAT1 was measured through the application of Applied Biosystems 7500 Fast. The comparative analysis of each gene was evaluated through a double standard curve, while the relative expression was analyzed using the 2^−ΔΔ^Ct method. In this method, ^Δ^Ct stands for target gene Ct value − internal reference gene Ct value, while ^ΔΔ^Ct = transfection group ^Δ^Ct − control group ^Δ^Ct.

### 2.4. Cell Transfection

All cells were cultured in DMEM complete medium with 10% FBS, 100 g/mL streptomycin, and 100 U/mL penicillin at 37°C with 5% CO_2_. The culture medium was changed every 1-2 days, and cells were passaged when they reached 90% confluence. The cells were grown on six-well plates for 24 h, and transfection was carried out as per the manufacturer's instructions once the cells reached 70% confluence. Subsequently, the cells were cultured for 48 h and collected for assessments.

### 2.5. MTT Assay

Cells at the logarithmic stage were grown in the 96-well plates and transfected when the cells reached 50% confluence. Each group was set with 3 multiple wells, and the transfected cells were cultured at different times. Furthermore, an MTT solution (Nanjing Senbeijia Biotechnology Co., LTD, China) was added and incubated at 37°C for 4 h after the cells in each well were cultured for 24, 48, 72, and 96 h. Cells were collected from the 96-well plates and the supernatant was discarded. The cells were also treated with 150 *μ*L of dimethyl sulfoxide (Shaanxi Tangyao Biological Technology Co., LTD, China) and swirled meticulously for 10 s to stop the reaction. Eventually, the optical density (OD value) of each well was determined using a microplate reader (Meigu Molecular Instruments (Shanghai) Co., LTD, China).

### 2.6. Colony Formation Assay

After centrifugation and trypsin digestion of the conventionally cultured cells, the supernatant was discarded, and the cell concentration was set at 1,000 cells/mL. Plates containing six wells were uniformly seeded with the prepared single-cell suspension and placed in an incubator for culture. The medium was replaced every 3 d, and the number of cell colonies was monitored 14 d later. Then, the cell colonies were fixed with 4% paraformaldehyde (Merck Chemical Technology (Shanghai) Co., Ltd., China), rinsed with PBS buffer 3 times, dyed with 1% crystal violet (Beijing Solaibao Technology Co., LTD, China) for 10 min, photographed, and counted.

### 2.7. Luciferase Reporter Assay

The results of prediction through an online bioinformatics computer program showed that there existed a continuous targeted site of binding between miR-342-3p and lncRNA NEAT1. According to the predicted results, the sequences of the binding site and mutation site on lncRNA NEAT1 and miR-342-3p were amplified and constructed into luciferase reporter vectors, which were denoted as lncRNA NEAT1Wt and lncRNA NEAT1 Mut luciferase recombinant vector plasmids. Co-transfection of logarithmic phase cells with a blank control mimic and miR-342-3p mimics into cells with lncRNA NEAT1WT or NEAT1 Mut luciferase recombinant vector plasmids was performed. The LipofectamineTM 2000 reagent was used to carry out the transfection procedure. The luciferase reporting kit (Shanghai Guchen Biological Co., LTD, China) was used to detect Renilla luciferase activity 48 h later. Renilla luciferase was used as an internal control to evaluate the relative activity of luciferase.

### 2.8. Western Blot

Cells were digested, centrifuged, and collected 48 h after transfection. By employing lysis buffer, the cells were lysed and then centrifuged. The supernatant containing total protein was retrieved and quantified through a BCA kit. After synthesizing a 10% SDS-PAGE gel, 60 *μ*g of protein was electrophoretically separated and then transferred to a PVDF membrane. The membrane was covered with 5% skim milk for 2 h. Additionally, the primary antibody was diluted according to the instruction of the antibody (1 : 300, Merck Chemical Technology (Shanghai) Co., Ltd., China). The membrane was added with the corresponding primary antibody and incubated overnight at 4°C on a shaker. Then, it was washed thrice with TBST solution and further incubated for 1 h with the secondary antibody (Merck Chemical Technology (Shanghai) Co., Ltd., China) at 1 : 3000 dilution at room temperature. Following rinsing the membrane, ECL solution (Yi Sheng Biotechnology (Shanghai) Co., LTD, China) was added to expose the membrane in a darkroom and the images were collected. GAPDH was employed as an internal reference, but the molecular weights of the target and internal reference were relatively close. In this western blot assay, primary antibody and secondary antibody remover were employed to wash the membrane to solve this problem.

### 2.9. Statistical Analysis

The experimental data were analyzed using SPSS 19.0. Mean ± standard deviation (*M* ± SD) was applied to summarize the collected data. Nonparametric rank-sum analysis was performed for nonnormally distributed data, while a *t*-test or analysis of variance (ANOVA) was used for normally distributed data. Moreover, the counting data were compared using chi-square analysis. *P* < 0.05 was considered significant for the complete data.

## 3. Results

### 3.1. Correlation of miR-342-3p and lncRNA NEAT1 Expression in CSCC Tissues

**Figure 1:**
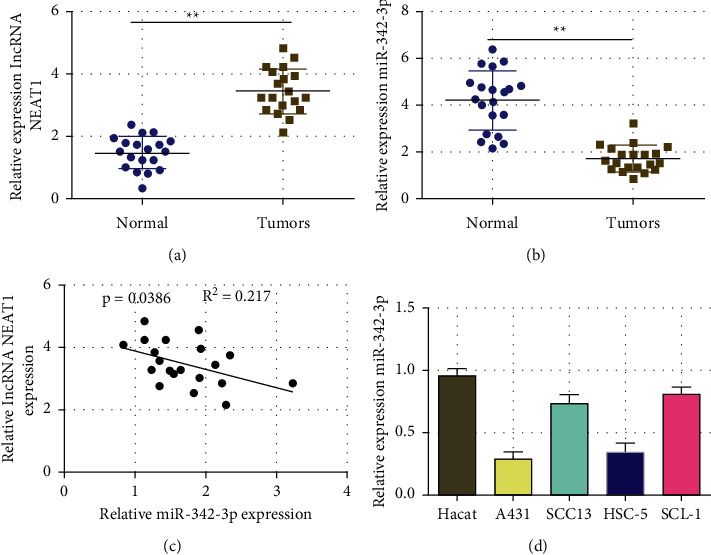
The qRT-PCR analysis on lncRNA NEAT1 and miR-342-3x expression in CSCC tissues. (a) The levels of lncRNA NEAT1 expression in CSCC and para-cancerous tissues of 20 cases; (b) the level of miR-342-3p in 20 cases of CSCC and para-cancerous tissues; (c) correlation analysis on the expression of miR-342-3p and lncRNA NEAT1 in CSCC; and (d) miR-342-3p expression in CSCC cell lines (SCL-1, HSC-5 ^*∗∗*^*P* <  0.01).

Our previous study demonstrated that lncRNA NEAT1 was overexpressed in CSCC tissues [11]. The expression of miR-342-3p and lncRNA NEAT1 in CSCC tissues was examined further in this work. The results illustrated a higher lncRNA NEAT1 expression (Figure 1(a)) and a lower miR-342-3p expression (Figure 1(b)) in the CSCC tissues than in the adjacent tissues. The expression of miR-342-3p and NEAT1 was negatively correlated in the CSCC based on correlation analysis (Figure 1(c)). The expression of lncRNA NEAT1 in skin squamous cell carcinoma cell lines (A431, SCC13, HSC-5, and SCL-1) and human normal skin cell line HaCaT was further detected by real-time quantitative PCR. The results showed that compared with the human normal skin cell line HaCaT, miR-342-3p was lowly expressed in cutaneous squamous cell carcinoma cells, and the lowest expression was in A431 and HSC-5 cells.

### 3.2. LncRNA NEAT1 Regulates the miR-342-3p Expression as a ceRNA in CSCC Cells

**Figure 2:**
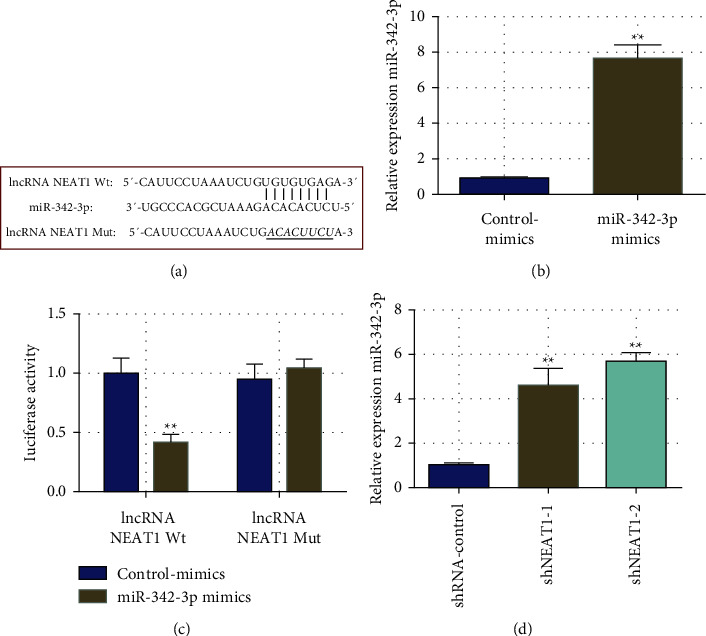
As a ceRNA, the lncRNA NEAT1 modulates the miR-342-3p expression. (a) Prediction of miR-342-3p-lncRNA NEAT1 binding site and mutant vector construction; (b) construction of miR-342-3p overexpressing cell lines; (c) luciferase reporter gene assessment verified the binding activity between miR-342-3p and lncRNA NEAT1; (d) changes in lncRNA NEAT1 expression level upon intracellular miR-342-3p knockdown. ^*∗∗*^*P* <  0.01, ^*∗*^*P* <  0.05.

The starBase bioinformatics tool identified the downstream miRNAs controlled by lncRNA NEAT1. It was identified that miR-342-3p and the lncRNA NEAT1 shared a similar binding site. As a result, mutant vectors were created (Figure 2(a)). The synthetic MiR-342-3p mimics were produced to transfect A431 cells. Consequently, these transfected miR-342-3p mimics significantly increased miR-342-3p expression in cells as compared to the control (control-mimics) cells (Figure 2(b)). Moreover, the luciferase reporter gene assessment showed that transfection with miR-342-3p mimics significantly reduced luciferase activity in the lncRNA NEAT1 Wt group compared to the control-mimics group, while no effect on luciferase activity in the lncRNA NEAT1 Mut group (Figure 2(c)). The lncRNA NEAT1 knockdown lentiviral vectors were enveloped. It was found that knocking down intracellular lncRNA NEAT1 (shNEAT1-1 and shNEAT1-2) significantly increased the amount of miR-342-3p expression in A431 cells (shRNA-control) as compared to a blank control. These findings indicated that the lncRNA NEAT1 acted as a ceRNA and regulated the miR-342-3p expression in CSCC cells.

### 3.3. MiR-342-3p Affects CSCC Cell Proliferation

**Figure 3:**
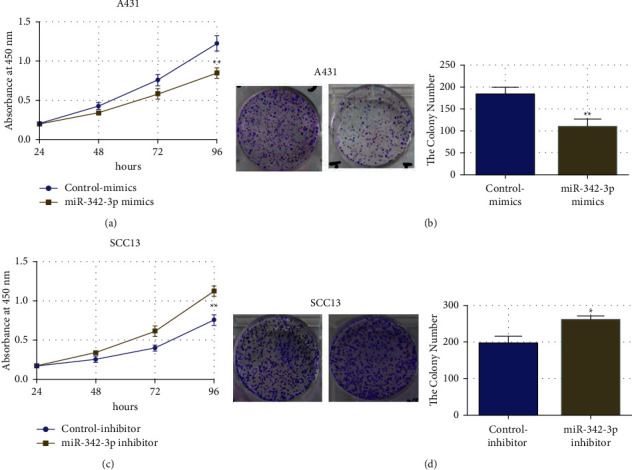
The effect of miR-342-3p on CSCC cell growth. ((a), (b)) the impact of miR-342-3p overexpression on A431 cell colony formation and proliferation; ((c), (d)) the effects of miR-342-3p inhibition on SCC13 cell colony formation and proliferation. ^*∗∗*^*P* <  0.01, ^*∗*^*P* <  0.05.

The plate colony formation assays and MTT assay were used to evaluate the impact of miR-342-3p overexpression or inhibition on CSCC cell proliferation. In contrast, miR-342-3p mimic transfection in A431 cells drastically decreased CSCC cell growth and colony formation compared to the control-mimics (Figures 3(a) and 3(b)). The miR-342-3p inhibitor transfection into SCC13 cells significantly improved CSCC cell proliferation and colony formation as compared to control-inhibitor-transfection (Figures 3(c) and 3(d)). These results indicated that miR-342-3p substantially affects CSCC cell growth.

### 3.4. LncRNA NEAT1 Regulates CUL4B Expression via miR-342-3p

**Figure 4:**
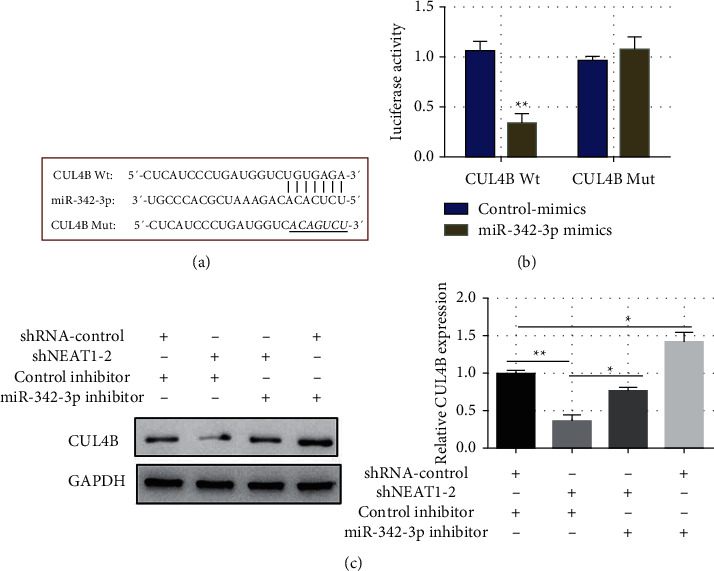
The lncRNA NEAT1 controls CUL4B expression through miR-342-3p. (a) Prediction of the miR-342-4p target gene CUL4B binding site and mutation site; (b) luciferase reporter gene analysis on miR-342-4p and CUL4B binding activity; and (c) the effect of lncRNA NEAT1 knockdown and miR-342-3p inhibition on CUL4B expression. ^*∗∗*^*P* <  0.01, ^*∗*^*P* <  0.05.

By binding to the 3′ untranslated region (UTR) of downstream mRNA, miRNA significantly controls the translation of downstream target genes and thereby performs biological activities. For the prediction of miR-342-3p downstream targets, bioinformatics tools (starBase and TargetScanHuman 7.1) were used. It was found that miR-342-3p binds to CUL4B (Figure 4(a)). CUL4B belongs to the Cullin family, whose members, as skeleton proteins, participate in the composition of Cullin-ring E3 ligases (CRLs), the largest class of ubiquitin ligase complexes in eukaryotes having a significant role in many pathophysiological processes by catalyzing polyubiquitination or monoubiquitination of substrate proteins. In the CUL4B Wt group, transfected miR-342-3p mimics substantially lowered the luciferase activity compared to the control-mimics group. However, in the CUL4B Mut group, transfection showed no significant impact on the luciferase activity (Figure 4(b)).

In A431 cells, knockdown of lncRNA NEAT1 (shNEAT1-2) significantly inhibited CUL4B expression compared with the blank control group (shRNA-control), and miR-342-3p inhibitor reversed CUL4B inhibition caused by lncRNA NEAT1 knockdown compared with the control-inhibitor (Figure 4(c)). These findings showed that lncRNA NEAT1 can regulate CUL4B expression through miR-342-3p.

### 3.5. LncRNA NEAT1 Regulates the Proliferation of CSCC Cells via miR-342-3p/CUL4B Signaling Axis

**Figure 5:**
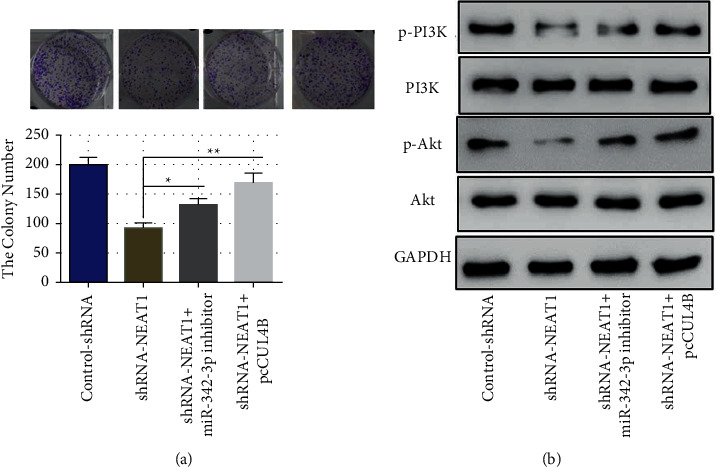
The miR-342-3p/CUL4B/PI3K-Akt signaling axis coordinates with lncRNA NEAT1 to control the proliferation of CSCC cells. (a) The effect of lncRNA NEAT1 on proliferation through miR-342-3p/CUL4B signaling axis detected by colony formation assay; (b) lncRNA NEAT1 affects the PI3K-Akt signaling pathway in CSCC cells via miR-342-3p/CUL4B signaling axis. ^*∗*^*P* <  0.05, ^*∗∗*^*P* <  0.01.

We also investigated if lncRNA NEAT1 affected CSCC cell proliferation through miR-342-3p/CUL4B signaling axis. The colony formation experiment revealed that the shNEAT1 + miR-342-3p inhibitor effectively reversed the inhibitory effect of lncRNA NEAT1 knockdown on cell proliferation as compared to the shNEAT1 group. The inhibitory effect of lncRNA NEAT1 knockdown was reversed in the shNEAT1 + pcCUL4B group compared with the shNEAT1 group (Figure 5(a)). It was reported that CUL4B can regulate the PI3K/Akt signaling pathway. Western blot analysis showed that lncRNA NEAT1 knockdown significantly reduced the expression of phosphorylated PI3K and Akt, but did not affect the expression of total PI3K and Akt compared with control shRNA. Compared with the shNEAT1 group, the shNEAT1 + pcCUL4B group significantly reversed the inhibitory influence of lncRNA NEAT1 knockdown on intracellular phosphorylated PI3K and Akt (Figure 5(b)). These results revealed that NEAT1 promotes CSCC cell proliferation through the miR-342-3p/CUL4B/PI3K-Akt signaling axis.

## 4. Discussion

LncRNA has more than 200 nucleotides with no protein-coding function. They are primarily involved in biological processes such as cell proliferation and apoptosis at the posttranscriptional, genetic, and transcriptional levels. They can bind to DNA, RNA, and protein and are associated with several types of malignancies, including CSCC [12, 13]. In CSCC, abnormal lncRNA expression was found to be able to serve as a potential marker for early tumor diagnosis, prognosis, and targets for therapy. Therefore, it is important to investigate lncRNAs for the treatment of CSCC [14, 15]. In this study, we demonstrated that the expression of lncRNA NEAT1 and miR-42-3p was negatively correlated. The lncRNA NEAT1, as a ceRNA, influenced CSCC cell proliferation through the miR-342-3p/CUL4B/PI3K-Akt signaling pathway. Therefore, lncRNA NEAT1 could be a biological marker or target for the diagnosis or treatment of CSCC.

A plethora of investigations has proved that lncRNA participates in the pathological process of CSCC development. LncRNA can regulate the concentration of intracellular miRNAs as a ceRNA, thus regulating the expression of its downstream target mRNA [16]. Different lncRNAs have been found to be overexpressed in various tumors, hence they could be used as novel targets or biomarkers for the diagnosis and treatment of CSCC. For example, lncRNA PICSAR is upregulated in CSCC tumors and can be used as a noninvasive biomarker for the diagnosis and prognosis of CSCC patients [17]. In addition, the upregulation of LINC00319 expression is strongly linked to poor prognosis in CSCC patients, providing some evidence as an alternative target for CSCC therapy [18]. In the current work, lncRNA NEAT1 was overexpressed, while miR-42-3p was poorly expressed in CSCC tissues, indicating a significant negative correlation between the two. Bioinformatics assessments and luciferase reporter gene analysis demonstrated that the lncRNA NEAT1 can regulate the miR-342-3p expression as a ceRNA.

MiRNA-342-3p performs an essential task in the occurrence and progression of a wide spectrum of tumors. In the case of hepatocellular carcinoma, for instance, the miR-342-3p expression was elevated which may hamper cell proliferation, mortality, and colony formation. The AAV-miR-342-3p treatment observed through *in vivo* analysis significantly reduced the tumor growth rate and increased the percentage of patients' overall survival [19]. Targeting AGR2 with miR-342-3p results in an inhibition of cell proliferation and migration in non-small-cell lung cancer (NSCLC) [20]. In addition, serum miR-342-3p is a prognostic and diagnostic biomarker for NSCLC [21]. It was confirmed that miR-342-3p inhibition or overexpression can affect CSCC cell proliferation. Meanwhile, bioinformatics evaluations and luciferase reporter assessment proved that miR-342-3p is capable of binding to CUL4B. CUL4B is a scaffold protein that is overexpressed in various solid malignancies and silences tumor suppressors by posttranscriptional means. For example, CUL4B is overexpressed in gastric cancer, and its overexpression is associated with poor prognosis and lymph node metastasis. CUL4B promotes gastric cancer cell invasion and epithelial-mesenchymal transition *in vitro* as well as tumor development and metastasis *in vivo* [22]. CUL4B is an independent risk factor for overall survival and disease-free survival in ovarian cancer. In vitro explorations have shown that CUL4B overexpression promotes tumor proliferation, while CUL4B knockdown significantly inhibits the proliferation of ovarian cancer cells [23].

The PI3K/AKT signaling pathway is one of the crucial signaling transduction pathways in cells. AKT is one of the downstream target molecules of PI3K and is also the core factor in this pathway. It can be activated by phosphorylation and affect the expression of downstream proliferation-related molecules, thus regulating the biological process of cell proliferation. It is tightly relevant to the occurrence and progression of CSCC [24, 25]. As an example, miRNA-451a blocks the progression of CSCC by means of the PI3K/AKT signaling pathway mediated through 3-phosphoinositol-dependent protein kinase-1 [26]. The long noncoding RNA LINC00520 inhibits CSCC development by inhibiting PI3K/Akt signaling and downregulating EGFR [27]. In this work, it was shown that lncRNA NEAT1 knockdown considerably reduced PI3K and Akt protein phosphorylation, but miR-342-3p inhibitor greatly reduced the repressive effect of lncRNA NEAT1 blocking on Akt and PI3K phosphorylation.

Taken together, the findings of this study demonstrated the negative correlations between the miR-42-3p and lncRNA NEAT1 expression in CSCC. This study is the first to demonstrate that the lncRNA NEAT1, as a ceRNA, affects the proliferation of skin squamous cell carcinoma cells through the miR-342-3p/CUL4B/PI3K-Akt signaling pathway. Therefore, lncRNA NEAT1 could be a biological marker or target for the diagnosis or treatment of CSCC. We will further investigate the clinical value of lncRNA NEAT1 as a diagnostic and prognostic marker for CSCC in future studies.

## Data Availability

The data are available from the corresponding author (jjf1971@ntu.edu.cn).

## References

[1] Ahmed B., Qadir M. I., Ghafoor S. (2020). Malignant melanoma: skin cancer-diagnosis, prevention, and treatment. *Critical Reviews in Eukaryotic Gene Expression*.

[2] Ke Y., Wang X. J. (2021). TGF*β* signaling in photoaging and UV-induced skin cancer. *Journal of Investigative Dermatology*.

[3] Suozzi K., Turban J., Girardi M. (2020). Cutaneous photoprotection: a review of the current status and evolving strategies. *Yale Journal of Biology & Medicine*.

[4] Que S. K. T., Zwald F. O., Schmults C. D. (2018). Cutaneous squamous cell carcinoma: management of advanced and high-stage tumors. *Journal of the American Academy of Dermatology*.

[5] Corchado-Cobos R., García-Sancha N., González-Sarmiento R., Pérez-Losada J., Cañueto J. (2020). Cutaneous squamous cell carcinoma: from biology to therapy. *International Journal of Molecular Sciences*.

[6] McCabe E. M., Rasmussen T. P. (2021). lncRNA involvement in cancer stem cell function and epithelial-mesenchymal transitions. *Seminars in Cancer Biology*.

[7] Peng W. X., Koirala P., Mo Y. Y. (2017). LncRNA-mediated regulation of cell signaling in cancer. *Oncogene*.

[8] Yu X., Li Z., Zheng H., Chan M. T. V., Wu W. K. K. (2017). NEAT1: a novel cancer-related long non-coding RNA. *Cell Proliferation*.

[9] Liu X., Yao W., Xiong H., Li Q., Li Y. (2020). LncRNA NEAT1 accelerates breast cancer progression through regulating miR-410-3p/CCND1 axis. *Cancer Biomarkers*.

[10] Zhang M., Weng W., Zhang Q. (2018). The lncRNA NEAT1 activates Wnt/*β*-catenin signaling and promotes colorectal cancer progression via interacting with DDX5. *Journal of Hematology & Oncology*.

[11] Gong Z., Shen G., Huang C., Zhang J., Ji J. (2022). Downregulation of lncRNA NEAT1 inhibits the proliferation of human cutaneous squamous cell carcinoma in vivo and in vitro. *Annals of Translational Medicine*.

[12] Bhan A., Soleimani M., Mandal S. S. (2017). Long noncoding RNA and cancer: a new paradigm. *Cancer Research*.

[13] Bridges M. C., Daulagala A. C., Kourtidis A. (2021). LNCcation: lncRNA localization and function. *The Journal of Cell Biology*.

[14] Piipponen M., Nissinen L., Kähäri V. M. (2020). Long non-coding RNAs in cutaneous biology and keratinocyte carcinomas. *Cellular and Molecular Life Sciences*.

[15] Kaushik S. B., Kaushik N. (2016). Non-coding RNAs in skin cancers: an update. *Non-Coding RNA Research*.

[16] Wang Y., Sun B., Wen X. (2020). The roles of lncRNA in cutaneous squamous cell carcinoma. *Frontiers in Oncology*.

[17] Lv J., Zhang W., Wang Y. (2021). Long non-coding RNA PICSAR serves as a non-invasive biomarker for the diagnosis and prognosis of cutaneous squamous cell carcinoma. *Clinical and Experimental Medicine*.

[18] Li F., Liao J., Duan X., He Y., Liao Y. (2018). Upregulation of LINC00319 indicates a poor prognosis and promotes cell proliferation and invasion in cutaneous squamous cell carcinoma. *Journal of Cellular Biochemistry*.

[19] Komoll R. M., Hu Q., Olarewaju O. (2021). MicroRNA-342-3p is a potent tumour suppressor in hepatocellular carcinoma. *Journal of Hepatology*.

[20] Xue X., Fei X., Hou W., Zhang Y., Liu L., Hu R. (2018). miR-342-3p suppresses cell proliferation and migration by targeting AGR2 in non-small cell lung cancer. *Cancer Letters*.

[21] Qin Y., Zhou X., Huang C. (2018). Serum miR-342-3p is a novel diagnostic and prognostic biomarker for non-small cell lung cancer. *International Journal of Clinical and Experimental Pathology*.

[22] Qi M., Jiao M., Li X. (2018). CUL4B promotes gastric cancer invasion and metastasis-involvement of upregulation of HER2. *Oncogene*.

[23] Duan P. J., Zhao J. H., Xie L. L. (2020). Cul4B promotes the progression of ovarian cancer by upregulating the expression of CDK2 and CyclinD1. *Journal of Ovarian Research*.

[24] Di Nardo L., Pellegrini C., Di Stefani A. (2020). Molecular genetics of cutaneous squamous cell carcinoma: perspective for treatment strategies. *Journal of the European Academy of Dermatology and Venereology*.

[25] Mercurio L., Albanesi C., Madonna S. (2021). Recent updates on the involvement of PI3K/AKT/mTOR molecular cascade in the pathogenesis of hyperproliferative skin disorders. *Frontiers of Medicine*.

[26] Fu J., Zhao J., Zhang H., Fan X., Geng W., Qiao S. (2020). MicroRNA-451a prevents cutaneous squamous cell carcinoma progression via the 3-phosphoinositide-dependent protein kinase-1-mediated PI3K/AKT signaling pathway. *Experimental and Therapeutic Medicine*.

[27] Mei X. L., Zhong S. (2019). Long noncoding RNA LINC00520 prevents the progression of cutaneous squamous cell carcinoma through the inactivation of the PI3K/Akt signaling pathway by downregulating EGFR. *Chinese Medical Journal*.

